# Standard error of measurement and smallest detectable change of the Sarcopenia Quality of Life (SarQoL) questionnaire: An analysis of subjects from 9 validation studies

**DOI:** 10.1371/journal.pone.0216065

**Published:** 2019-04-29

**Authors:** Anton Geerinck, Vidmantas Alekna, Charlotte Beaudart, Ivan Bautmans, Cyrus Cooper, Fabiana De Souza Orlandi, Jerzy Konstantynowicz, Beatriz Montero-Errasquín, Eva Topinková, Maria Tsekoura, Jean-Yves Reginster, Olivier Bruyère

**Affiliations:** 1 World Health Organization Collaborating Center for Public Health Aspects of Musculoskeletal Health and Ageing, Department of Public Health, Epidemiology and Health Economics, University of Liège, Liège, Belgium; 2 Faculty of Medicine, Vilnius University, Vilnius, Lithuania; 3 Frailty in Aging Research Group, Vrije Universiteit Brussel, Brussels, Belgium; 4 MRC Environmental Epidemiology Unit, Southampton General Hospital, Southampton, United Kingdom; 5 Department of Gerontology, Federal University of São Carlos, São Carlos, SP, Brazil; 6 Department of Pediatric Rheumatology, Immunology and Metabolic Bone Diseases, Medical University of Bialystok, Bialystok, Poland; 7 Department of Geriatrics, University Hospital Ramón y Cajal, Madrid, Spain; 8 Department of Geriatrics and Gerontology, 1st Faculty of Medicine, Charles University, Prague, Czech Republic; 9 Department of Physical Therapy, School of Health and Welfare, Technological Institute of Western Greece, Aigio, Greece; Iranian Institute for Health Sciences Research, ISLAMIC REPUBLIC OF IRAN

## Abstract

**Objectives:**

The Sarcopenia Quality of Life (SarQoL) questionnaire, a sarcopenia-specific patient-reported outcome measure, evaluates quality of life with 55 items. It produces 7 domain scores and 1 overall quality of life score, all between 0 and 100 points. This study aims to contribute to the interpretation of the SarQoL scores by calculating the standard error of measurement (SEM) and smallest detectable change (SDC) in a sample of subjects from 9 studies.

**Methods:**

Subjects from 9 studies (conducted in Belgium, Brazil, Czech Republic, England, Greece, Lithuania, Poland and Spain) were included. The SEM, a measure of the error in the scores that is not due to true changes, was calculated by dividing the standard deviation of the difference between test and retest scores (SDdiff) by √2. The SDC, defined as change beyond measurement error, was calculated by multiplying SDdiff by 1.96. Bland-Altman plots were assessed for the presence of systematic errors.

**Results:**

A total of 278 sarcopenic subjects, aged 77.67 ± 7.64 years and 61.5% women, were included. The SEM for the overall SarQoL score ranged from 0.18 to 4.20 points for the individual studies, and was 2.65 points when all subjects were analyzed together. The SDC for the overall score ranged from 0.49 to 11.65 points for the individual studies, and was 7.35 points for all subjects. The Bland-Altman plots revealed no systematic errors in the questionnaire.

**Conclusion:**

This study shows that, for individual subjects, a change in overall quality of life of at least 7.35 points (on a scale from 0 to 100) would have to be observed to confirm that a true change, beyond measurement error, has occurred. It also demonstrated that the SarQoL questionnaire is a precise instrument, with the observed scores within less than 3 points of the theoretical “true score”.

## Introduction

Sarcopenia, often described as the age-related loss of muscle mass and strength, and defined by the European Working Group on Sarcopenia in Older People (EWGSOP2) as *“a progressive and generalised skeletal muscle disorder that is associated with increased likelihood of adverse outcomes including falls*, *fractures*, *physical disability and mortality”*, has been the subject of increased scientific attention as its prevalence and consequences have become more known [1]. Sarcopenia is confirmed to be present when a patient is diagnosed with low muscle strength and low muscle mass. When low physical performance is also established, that person is diagnosed with severe sarcopenia [1]

A systematic review conducted in 2014 which estimated the prevalence of sarcopenia diagnosed with the EWGSOP-algorithm in older community-dwelling adults found a range of 1 to 29% (up to 30% in women), while a recent meta-analysis which included 35 articles and a total of 58404 healthy subjects aged 60 years and older found an overall prevalence of sarcopenia of 10% (95% CI: 8–12%) in men and 10% (95% CI: 8–13%) in women diagnosed with the EWGSOP, the International Working Group on Sarcopenia (IWGS) or the Asian Working Group for Sarcopenia (AWGS) definitions [2,3]. It should be mentioned that the prevalence of sarcopenia varies greatly depending on the definition used, as demonstrated by Beaudart et al., who applied 6 different diagnostic criteria for sarcopenia to a single cohort of subjects and found a prevalence rate from 4.39% to 32.8% [4].

Projections about the future prevalence of sarcopenia (as diagnosed by the EWGSOP-criteria) in the European Union (EU28) predict a rise from 10.9 million people in 2016 to 18.7 million in 2045 on the low end and from 19.7 million to 32.3 million people on the high end [5]. Sarcopenia is a major public health problem and its impact will continue to grow, which should incite policy makers to act.

The available evidence concerning the impact and association of sarcopenia with several health outcomes has been steadily growing during the last decade. A systematic review and meta-analysis published in 2017 provided a comprehensive summary of what is currently known on the subject. This review included 17 prospective studies in which sarcopenia was diagnosed according to the EWGSOP guidelines. The authors found a higher risk for mortality (OR = 3.596; 95% CI = 2.96–4.37) and functional decline (OR = 3.03; 95% CI = 1.80–5.12) as well as a higher rate of falls and a higher incidence of hospitalization. The evidence on the incidence of fractures and the length of hospital stay was inconclusive [6].

The subject of quality of life in sarcopenia has mostly been examined using generic questionnaires such as the Short-Form 36-Item (SF-36) and the EuroQoL 5-Dimension (EQ-5D) [7]. Recently, a new instrument, the Sarcopenia Quality of Life (SarQoL) questionnaire has become available. It is specifically designed to measure quality of life in sarcopenic, community-dwelling individuals aged 65 years or older and was developed in 2013–2015 by Beaudart et al. [8]. It has, to date, been translated into more than 20 languages [8].

The psychometric properties of the SarQoL questionnaire have been evaluated and published for 6 language-versions: the original questionnaire in French, and the English, Dutch, Polish, Romanian and Greek translations [9–14]. These examined the discriminative power, internal consistency, construct validity, test-retest reliability and the presence of floor or ceiling effects. These 6 studies found that the questionnaire can discriminate between sarcopenic and non-sarcopenic participants, with the former having significantly lower scores for the 7 domains and the overall score, and that the questionnaire possesses good internal consistency (Cronbach’s alpha of 0.87, 0.88, 0.95, 0.92, 0.88 and 0.96). These studies also confirmed the construct validity of the SarQoL questionnaire with the help of hypotheses on correlations between the questionnaire and the SF-36 and EQ-5D, and demonstrated that the SarQoL questionnaire has an excellent test-retest reliability (intraclass correlation coefficient/ICC = 0.91, 0.95, 0.99, 0.98 and 0.96) [9–14]. Lastly, floor and ceiling effects were absent from all 6 published validation studies [9–14]. These results provide convincing evidence for the validity and reliability of the SarQoL questionnaire for the evaluation of quality of life in sarcopenic, community-dwelling older people.

However, until now, the standard error of measurement (SEM) and the smallest detectable change (SDC) of the SarQoL questionnaire have not yet been calculated. These parameters supply important information on the reliability of the instrument in question by indicating the range in which the theoretical “true” score lies; and supply context when interpreting data from longitudinal measurements by indicating by how much the score needs to change before one can be reasonably certain that a true change has occurred. Clinicians and researchers could use the values for SEM and SDC as a yardstick in the interpretation of the SarQoL scores, whether obtained in clinical practice or as part of a research project. The results of this study should prove particularly valuable in the interpretation of data from interventional clinical trials, and will hopefully expedite the adoption of this PROM in clinical trials [15].

The primary objective of this study is to determine the SEM and SDC of the SarQoL questionnaire in a sample of subjects from 9 international validation studies. The secondary objectives are to examine the measurement error of the questionnaire with the help of a Bland-Altman analysis, and to update the results previously obtained for the test-retest reliability of the SarQoL questionnaire in the complete sample.

## Material and methods

This study combined data from 9 cohorts in 8 different countries that were established to test the psychometric properties of the SarQoL questionnaire after translation into the local language. The team behind the SarQoL questionnaire have made a concerted effort to widen the reach of the questionnaire by having it translated into a multitude of languages. To accomplish this, they have partnered with researchers from a host of countries and language groups, who were able and willing to undertake a translation of the questionnaire. The local teams responsible for the translations were also encouraged to carry out a validation study of the translation they produced, if feasible. A considerable number of them undertook this effort, although not all validations have been published. The researchers from 9 validation studies that had the necessary data for the current analysis were contacted and agreed to share their data. All the included studies obtained approval from their local ethics committees, and written informed consent from their participants.

### Population

Subjects were included in the 9 validation studies if they were 60 years of age or older and community-dwelling. For this analysis, we included all subjects who were diagnosed as being sarcopenic, who completed the SarQoL questionnaire twice and reported that their health had been stable in the interval between the two administrations.

### The SarQoL questionnaire

The analyses in this article center around the test-retest data for the SarQoL questionnaire collected by the 9 included studies. The SarQoL questionnaire is a patient-reported outcome measure (PROM) designed specifically for use with sarcopenic, community-dwelling subjects 65 years of age or older. The questionnaire consists of 55 items distributed over 22 questions, with the items categorized into 7 domains of health-related quality of life (HRQoL). These domains are: “Physical and Mental Health” (D1), “Locomotion” (D2), “Body Composition” (D3), “Functionality” (D4), “Activities of Daily Living” (D5), “Leisure activities” (D6), and “Fears” (D7). Apart from the domain scores, an Overall score for quality of life is also calculated. All scores are situated on a scale from 0 to 100, with 0 being the worst possible quality of life, and 100 the best possible. The questionnaire is auto-administered and takes about 10 minutes to complete [9]. More information on the SarQoL questionnaire and the different language-specific versions can be found on www.sarqol.org.

### Test-retest reliability

The test-retest reliability of a questionnaire quantifies the extent to which a questionnaire produces the same scores during repeated measurements, provided that the participants’ health remains stable. It is measured by the intraclass correlation coefficient (ICC) under a 2-way mixed model with absolute agreement specified, and its associated 95% confidence interval. A questionnaire is considered reliable if the obtained ICC values are greater than 0.70 [16].

### Standard error of measurement

The standard error of measurement has been defined as *“the determination of the amount of variation or spread in the measurement errors for a test”* [17]. The SEM is considered to be a parameter for the amount of measurement error present in an instrument, and is subsequently an indicator of the reliability of said instrument. Much like the interpretation of the standard deviation around the mean value, the SEM can be used to provide a range around the observed value within which the theoretical “true” value lies. The interval between plus and minus 1 SEM provides a probability of 68% of containing the true value. For ± 2 SEM the probability becomes 95% and for ± 3 SEM we end up with 99% probability.

### Smallest detectable change

The smallest detectable change is defined as the change in the instrument’s score beyond measurement error [18]. This means that the SDC provides a value for the minimum change that needs to be observed in order to be confident that the observed change is real and not, potentially, a product of measurement error in the instrument. The SDC can be calculated for individual subjects (SDC_ind_) as well as for comparisons of mean scores between groups (SDC_group_) [18]. Both provide utility: The SDC_ind_ can be used in clinical practice or to label individual subjects in a study sample as either changed or unchanged. The SDC_group_ provides an aid to the interpretation of mean scores of groups. This can lend greater credibility to the results of interventional trials that use the SarQoL questionnaires, and that want to know whether quality of life has changed in the intervention and control group as a whole.

### Bland-Altman analysis

The Bland-Altman plot provides a visual representation of the presence of systematic errors in an instrument. The Bland-Altman plot is based around three variables: the mean systematic difference between test and retest scores ($\bar{d}$), and the upper and lower limit of agreement, which span 95% of observations, assuming that the values for the difference between test and retest scores are distributed normally [18,19]. These variables are integrated into a scatter plot where the difference between test and retest values is put on the Y-axis and the average of the test and retest values is put on the X-axis.

### Statistical analysis

Data were analyzed using IBM SPSS Statistics, version 24.0.0.0 for Windows (Armonk, NY: IBM Corp). The distribution of the variables was determined by examining the histogram, the quantile-quantile-plot, the Shapiro-Wilk test and the difference between mean and median. Variables that are normally distributed are reported as mean ± standard deviation and non-normal variables as median (25^th^ percentile– 75^th^ percentile). Nominal variables are reported as absolute (n) and relative frequencies (%).

Differences between groups with regards to clinical characteristics were examined with one-way anova analysis for continuous variables and chi-squared test for nominal variables.

The SEM was calculated by first creating a variable for the difference between the score obtained during the first and the second administration (test score—retest score = *Difference*). Next, we calculated the standard deviation of *Difference* in our sample (SD_*difference*_) and divided the obtained value by the square root of 2 (SEM = $\frac{SDdifference}{\surd 2}$) [18,20].

The SDC_ind_ was calculated with the formula [SDC_ind_ = 1.96 * √2 * SEM], and the SDC_group_ was calculated by dividing the SDC_ind_ by the square root of the number of subjects in the sample ($\frac{SDCind}{\surd n}$) [18].

The ICC was calculated with a 2-way mixed model and absolute agreement specified.

The mean difference score ($\bar{d}$) was calculated by calculating the mean of the differences between test and retest scores for all subjects [Mean(test score—retest score)]. The 95% limits of agreement were calculated with the formula [$\bar{d}$ ± (1.96 * SD_*difference*_)] [18,21]. Bland-Altman plots were created in SPSS following the instructions given in IBM tech-note n° 19420 [22]

Results were considered significant at p≤0.05.

## Results

### Characteristics of included studies

Information on the diagnosis of sarcopenia and the characteristics of the test-retest administration are given in Table 1.

**Table 1:** Characteristics of included studies.

	Sarcopenia diagnosis				Time between test and retest administration	Mode of administration	
	Sarcopenia definition	Muscle mass assessment	Muscle strength assessment	Physical performance assessment		Test	Retest
Belgium (Dutch) [12]	EWGSOP	BIA	Martin-Vigorimeter	Gait speed	2 weeks	At study center	At home
Belgium (French) [9]	EWGSOP	DXA	Hand dynamometer	SPPB	2 weeks	At study center	At home
Brazil	EWGSOP	DXA	Hand dynamometer	Gait speed	2 weeks	At home	At home
Czech Republic [23]	FNIH	DXA	Hand dynamometer	SPPB	2 weeks	At home or at study center without staff present	At home or at study center without staff present
England [10]	EWGSOP	DXA	Hand dynamometer	Gait speed	2 weeks	At home	At home
Greece [14]	EWGSOP	BIA	Hand dynamometer	Gait speed	2 weeks	At study center	At study center
Lithuania	EWGSOP	DXA	Hand dynamometer	SPPB	2 weeks	At study center	At study center
Poland [13]	EWGSOP	Lee equation [24]	Hand Dynamometer	Not performed	2 weeks	At study center	At study center
Spain	FNIH	DXA	Hand dynamometer	SPPB	2 weeks	At study center	At home

EWGSOP: European Working Group on Sarcopenia in Older People; BIA: bioelectrical impedance analysis; DXA: dual-energy x-ray absorptiometry; FNIH: Foundation for the National Institutes of Health

### Clinical characteristics

The 278 participants included in the analysis had a mean age of 77.67 ± 7.64 years, ranging from 60 to 98 years old. The majority of subjects were women, namely 171 participants or 61.5% of the complete sample. The participants had a mean body mass index of 25.57 ± 4.40 kg/m^2^, spanning the whole gambit from underweight to morbidly obese with a minimum value of 17.42 kg/m^2^ and a maximum value of 46.10 kg/m^2^. In terms of prescription drug use, the subjects took on average 4.78 ± 2.71 drugs (range: 0–13), linked to the number of comorbidities which was 3.59 ± 2.01 (range: 0–11). Clinical characteristics are reported in Table 2.

**Table 2:** Clinical characteristics for individual studies–mean ± SD or n(%).

	All	Belgium (Dutch)	Belgium (French)	Brazil	Czech Republic	England	Lithuania	Greece	Poland	Spain
n	278	26	29	12	48	10	58	50	30	15
Age (years)	77.67 ± 7.64	81.00 ± 5.88	77.03 ± 6.58	70.75 ± 6.57	82.96 ± 6.05	78.90 ± 2.56	80.18 ± 6.42	72.10 ± 7.71	73.82 ± 7.06	77.60 ± 6.27
Gender										
Female	171 (61.5)	12 (46.2)	19 (65.5)	6 (50.0)	37 (77.1)	3 (30.0)	28 (48.3)	37 (74.0)	19 (63.3)	10 (66.7)
Body mass index (kg/m^2^)	25.57 ± 4.40	26.71 ± 4.75	23.16 ± 3.19	24.84 ± 4.32	29.16 ± 5.78	24.00 ± 2.73	24.62 ± 2.54	24.05 ± 3.39	27.01 ± 4.46	24.17 ± 1.99
Drugs (n)	4.78 ± 2.71	3.81 ± 2.62	6.72 ± 2.76	7.25 ± 1.55	6.27 ± 3.30	6.00 ± 2.45	4.36 ± 1.25	3.50 ± 1.28	2.70 ± 2.84	5.13 ± 2.75
Concomitant illnesses (n)	3.59 ± 2.01	2.48 ± 1.64	4.93 ± 2.36	4.17 ± 1.59	5.79 ± 1.47	NA	2.98 ± 0.78	2.96 ± 1.01	1.60 ± 1.85	3.80 ± 2.04

As expected, one-way anova analyses and chi-squared test revealed that the 9 studies differed significantly in terms of clinical characteristics. The results from these post-hoc analyses can be found in S1–S5 Tables.

The test-retest reliability of the SarQoL questionnaire in the complete sample resulted in an ICC of 0.969 (95% CI = 0.961–0.975) for the Overall score. Of the individual domains, 4 obtained an ICC higher than 0.9, namely domain 1, 2, 4 and 5, and all obtained ICC’s higher than 0.7. The detailed results for the test-retest reliability can be found in Table 3.

**Table 3:** Results for complete analysis (n = 278).

	Test scores	Retest scores	ICC (95% CI)	$\bar{d}$ (95% CI)	SD_diff_	SEM	SDC_ind_	SDC_group_	95% LoA
D1: Physical & mental health	56.56 ± 17.00	57.42 ± 17.12	0.915 (0.894; 0.933)	0.86 (0.04; 1.68)	6.98	4.94	13.68	0.82	-12.82; 14.54
D2: Locomotion	54.95 ± 21.40	54.88 ± 21.54	0.944 (0.929; 0.955)	-0.07 (-0.93; 0.78)	7.23	5.11	14.17	0.85	-14.24; 14.1
D3: Body composition	55.36 ± 16.91	56.10 ± 17.18	0.836 (0.797; 0.869)	0.74 (-0.41; 1.89)	9.74	6.89	19.09	1.14	-18.35; 19.83
D4: Functionality	62.31 ± 17.08	62.70 ± 16.61	(0.952 (0.939; 0.962)	0.39 (-0.23; 1.01)	5.24	3.71	10.27	0.62	-9.88; 10.66
D5: Activities of daily living	55.55 ± 17.33	55.40 ± 17.73	0.915 (0.894; 0.933)	-0.15 (-1.00; 0.70)	7.23	5.11	14.17	0.85	-14.32; 14.02
D6: Leisure activities	37.61 ± 17.83	37.00 ± 19.23	0.754 (0.698; 0.800)	-0.59 (-2.13; 0.94)	13.04	9.22	25.56	1.53	-26.15; 24.97
D7: Fears	78.98 ± 17.47	78.96 ± 17.13	0.783 (0.733; 0.825)	-0.02 (-1.37; 1.33)	11.42	8.08	22.38	1.34	-22.4; 22.36
Overall score	57.71 ± 14.97	57.89 ± 15.03	0.969 (0.961; 0.975)	0.18 (-0.26; 0.63)	3.75	2.65	7.35	0.44	-7.17; 7.53

ICC = intraclass correlation coefficient; $\bar{d}$ = mean difference score; CI = confidence interval; SD_diff_ = standard deviation of difference scores; SEM = standard error of measurement; SDC_ind_ = smallest detectable change for individual subject; SDC_group_ = smallest detectable change for group; LoA = limits of agreement

### Standard error of measurement

The SEM for the Overall score of the SarQoL questionnaire in the complete sample is 2.65 points. This means that one can be 68% confident (± 1 SEM) that the ‘true’ score of a subject can be found between -2.65 and +2.65 points from the observed score, and 95% confident (± 2 SEM) that the ‘true’ score is situated between -5.3 and +5.3 points of the observed score. The SEM for the different domains of the SarQoL questionnaire in the complete sample varied between 3.71 for domain 4 and 9.22 points for domain 6. The SEM-values for the complete sample can be found in Table 3, while the SEM-values for the individual included studies are available in Table 4.

**Table 4:** SEM and SDC for individual studies.

		Belgium (Dutch)	Belgium (French)	Brazil	Czech Republic	England	Lithuania	Greece	Poland	Spain
SEM	D1	6.57	6.50	3.69	7.02	9.48	0.54	3.04	2.61	3.08
	D2	6.13	8.26	3.63	6.91	2.89	0.68	4.41	1.19	5.14
	D3	7.81	10.59	1.70	10.05	6.37	1.57	7.09	1.69	4.14
	D4	3.75	5.75	3.16	4.65	4.77	0.53	3.82	1.97	3.28
	D5	7.38	8.07	2.45	4.65	6.30	0.54	6.36	2.92	2.51
	D6	14.70	12.98	7.09	10.68	12.14	0	7.29	0.00	7.52
	D7	16.26	20.85	0.00	5.72	7.74	2.50	10.12	3.23	4.05
	Overall	2.54	4.06	2.17	2.86	4.20	0.18	3.34	1.07	1.73
SDC _ind_	D1	18.21	18.30	10.24	19.45	26.28	1.49	8.41	7.23	8.54
	D2	16.99	22.79	10.07	19.15	8.00	1.89	12.22	4.67	14.26
	D3	21.71	29.21	4.71	27.86	17.65	4.35	19.65	7.43	11.46
	D4	16.15	10.40	8.75	12.89	13.23	1.47	10.60	5.46	9.10
	D5	20.46	22.27	6.79	12.89	17.47	1.51	17.62	8.11	6.96
	D6	40.76	35.98	19.64	29.61	33.65	0	20.22	0.00	20.85
	D7	45.07	29.43	0.00	15.85	21.45	6.94	28.05	8.95	11.21
	Overall	7.05	11.34	6.00	7.92	11.65	0.49	9.24	2.96	4.81
SDC _group_	D1	3.57	3.40	2.95	2.81	8.31	0.20	1.19	1.32	2.21
	D2	3.33	4.23	2.91	2.76	2.53	0.25	1.73	0.85	3.68
	D3	4.26	5.42	1.36	4.02	5.58	0.57	2.78	1.36	2.96
	D4	3.17	1.93	2.53	1.86	4.18	0.19	1.50	1.00	2.35
	D5	4.01	4.14	1.96	1.86	5.53	0.20	2.49	1.48	1.80
	D6	7.99	6.68	5.67	4.27	10.64	0	2.86	0.00	5.38
	D7	8.84	5.47	0.00	2.29	6.78	0.91	3.97	1.63	2.90
	Overall	1.38	2.11	1.73	1.14	3.68	0.06	1.31	0.54	1.24

SEM: standard error of measurement; SDC_ind_: smallest detectable change for individual subjects; SDC_group_: smallest detectable change for groups

### Smallest detectable change

The SDC_ind_ for the Overall score of the SarQoL questionnaire in the complete sample is 7.35 points. This means that the Overall quality of life score of an individual would have to change with at least 7.35 points (on a scale of 0 to 100) before the observed change can be considered to be a true change in the quality of life of a subject, and not potentially a result of measurement error. The SDC_ind_ for the 7 domains of the SarQoL questionnaire goes from a minimum value of 10.27 points for domain 4 to a maximum value of 25.56 points for domain 6. The SDC_group_ for the Overall score in the complete sample is 0.44 points. The SDC-values for the complete sample can be found in Table 3. The SDC-values for the individual included studies are available in Table 4.

### Bland-Altman analysis

**Fig 1:**
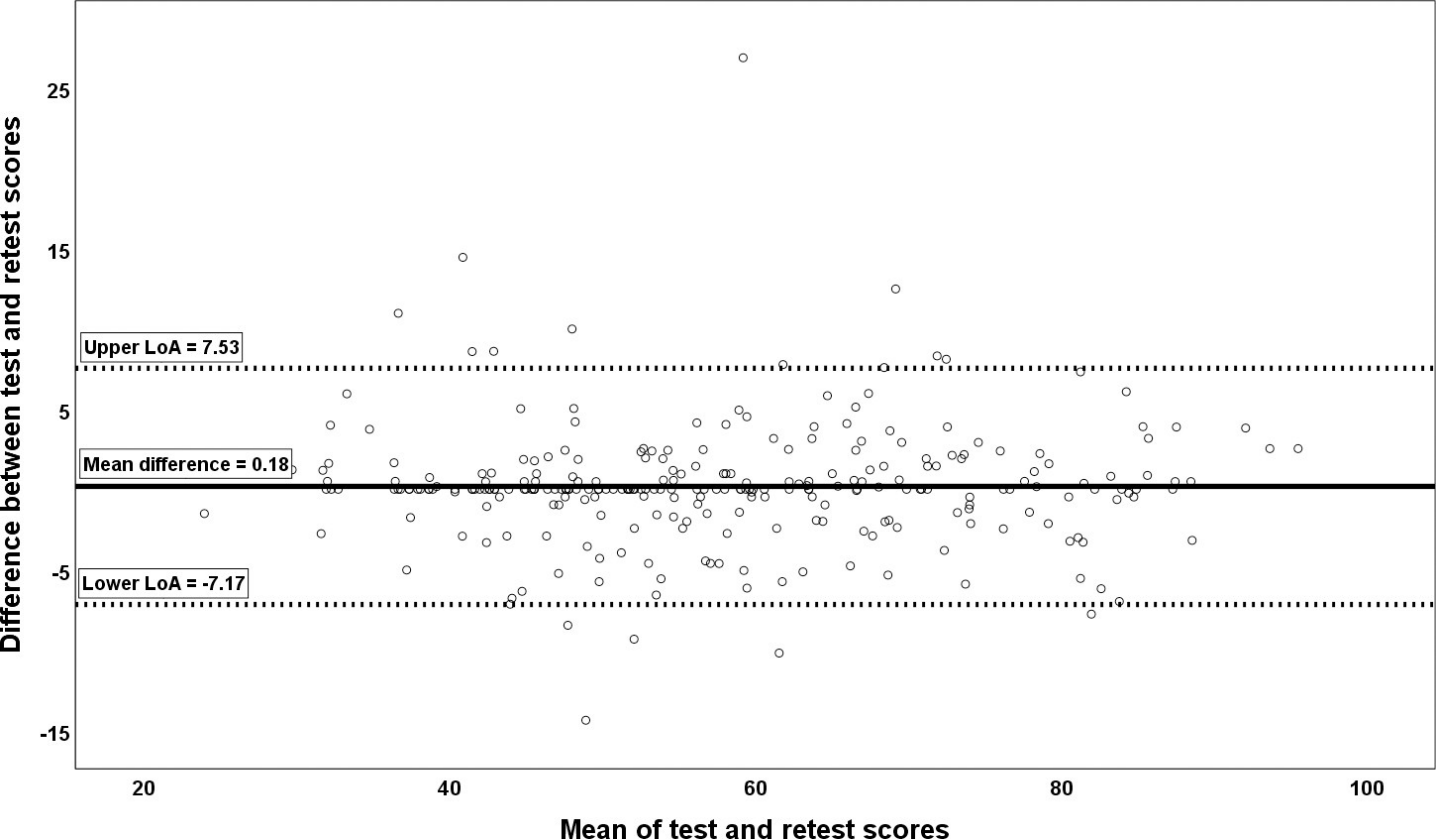
Bland Altman plot for the SarQoL overall score in the complete sample.

The mean difference score in the complete sample for the Overall score of the SarQoL questionnaire is 0.18 points (95% CI = -0.26; 0.63) which shows that there is no systematic bias between the two administrations of the questionnaire because the confidence interval contains zero. The mean difference scores in the complete sample for the 7 domains are not significant (95% CI contains zero) for domains 2, 3, 4, 5, 6 and 7, once again indicating the absence of systematic bias. One domain in the complete sample does have a small but significant mean difference score, namely domain 1 [0.86 points (0.04; 1.68)], indicating the presence of a very slight systematic error. The full results of the Bland-Altman analysis are detailed in Table 3. A Bland-Altman plot for the Overall score in the complete sample is provided as Fig 1.

## Discussion

In this study, values were obtained for the standard error of measurement and the smallest detectable change of the SarQoL questionnaire in a sample of 278 sarcopenic subjects hailing from 8 different countries and 9 different language-groups. The measurement error inherent to the questionnaire was found to be 2.65 points, and the minimum change needed to be confident that a real change in overall quality of life has occurred for an individual patient was 7.35 points. Systematic bias was further investigated with the method of Bland & Altman, and showed that there is no systematic bias for almost all domains (with domain 1 as the exception) and the overall score of the SarQoL questionnaire.

The SEM for the Overall score of the SarQoL questionnaire of 2.65 points represents 2.65% of the possible range of the Overall score (0–100) and 3.81% of the observed range of the SarQoL scores in the complete sample (min = 24.74; max = 94.22; range = 69.48).

This value for the standard error of measurement compares favorably with SEMs for the SF-36, the most frequently used quality of life questionnaire in sarcopenic populations. Hart found a SEM of 4 points for the Physical Component Summary (PCS–range: 0–100 points) and the Mental Component Summary (MCS–range: 0–100 points) of the SF-36 in a population of 68 subjects with a variety of orthopedic impairments [25] and Palmer calculated a SEM of 3.09 points for the PCS and 5.57 points for the MCS in a population of 233 subjects with joint hypermobility [26]. Other studies looked at the SEM for the 8 domains of the SF-36 (all range between 0–100 points), and found SEMs between 8.82 and 34.52 points in 106 women undergoing surgery for breast cancer [27], between 13.2 and 44.7 points in 92 subjects with neck pain [28], between 6.82 and 11.22 points for 628 subjects undergoing foot or ankle surgery [29], and between 11 and 32 points for 515 subjects undergoing orthopedic surgery [30]. While these have been calculated in populations that differ from ours, they show a trend for higher standard errors of measurement compared to the SarQoL questionnaire.

The SDC of the Overall score (7.35 points) of the SarQoL questionnaire is similar to the SDC found for the PCS and MCS of the SF-36. Palmer obtained SDCs of 8.56 points for the PCS and 15.44 points for the MCS, while Hart found SDCs of 9 points both for the PCS and MCS [25,26].

The results for the 7 domains of the SarQoL questionnaire in the complete sample show considerably higher SEM and SDC values compared to the Overall score. These values seem to correspond roughly to the number of items in each domain. When looking at the 3 domains with the least number of items (D6: 2 items; D3: 3 items; D7: 4 items), the largest SEM and SDC values are found, between 6.89 and 9.22 points for the SEM and between 19.09 and 25.51 points for the SDC. This contrasts with the 4 domains with larger numbers of items (D1: 8 items, D2: 9 items; D4: 14 items; D5: 15 items) which have SEM-values between 3.71 and 5.11 points and SDC-values between 10.27 and 14.17 points. It is not surprising that a domain score based on a larger number of items has greater precision and lower variability, represented by the standard deviation of the difference between test and retest scores.

The detailed breakdown of the SEM and SDC values obtained for the individual studies included in the analysis demonstrates the fact that the SEM and SDC depend on the population in which they are calculated. There is considerable variability between the studies, but not within the studies (i.e. studies with lower or higher SEM and SDC values are so for all the domains and the Overall score, and do not report low values for one domain and high for another). On the lower end are found the studies carried out in Lithuania, Poland and Spain, in the middle those carried out in Belgium (Dutch), Brazil and the Czech Republic and on the higher end those carried out in Greece, England and Belgium (French). We were unable to formulate convincing hypotheses that could begin to explain why certain studies reported lower or higher values for SEM and SDC based on the clinical or study characteristics. It is likely that the observed variation is just the manifestation of the fact that the SEM and SDC are specific to the population in which they have been measured.

The Bland-Altman analysis, detailed in Table 3 and visually represented for the Overall score in Fig 1, shows that a very small systematic bias exists in only one domain. It is unlikely that this systematic bias is clinically relevant because of its small confidence interval and the fact that the lower end of the interval is extremely close to zero (95% CI = 0.04; 1.68). These results mean that clinicians and researchers can have confidence when administering the questionnaire that the results will not be distorted by systematic bias.

The analysis of the test-retest reliability in the complete sample confirmed the results from previous validation studies. The significantly larger sample in the combined analysis means that the confidence intervals found are much narrower than has been obtained previously. These results should inspire confidence that the SarQoL questionnaire is a reliable instrument.

### Strengths and limitations

The main strength of this study is the fact that we were able to assemble a relatively large and heterogeneous sample (n = 278) of sarcopenic participants. This has the important advantage that the values calculated for the SEM and SDC are not dependent on a particular population, and could thus be more confidently used as a benchmark in future studies. The studies included in the analysis used different diagnostic criteria and instruments to establish sarcopenia. This is an advantage in this particular situation because the SEM and SDC values found in this study are not specific to a single definition of sarcopenia, but should be valid for different diagnostic criteria for sarcopenia, measured with different instruments. By combining multiple samples that differ with regards to clinical characteristics, we were able to find a middle ground and values for the SEM and SDC that are not highly specific to a single population. The sample size, which would be very difficult to gather in a single study, increased the accuracy of the standard deviation of the difference between test and retest score. Given that this parameter is key in the calculation of the SEM and SDC, the accuracy of these two parameters was enhanced by the large sample size. Because the SarQoL questionnaire has undergone validation in multiple languages, we were able to use test-retest data to calculate the SEM and the SDC, which is the preferred method because it takes into account biological variation, change of mood or concentration and other circumstances [18]. Since the data on which this study was based incorporates these elements and their subsequent influence on the SarQoL score, they have greater credibility than if other methods for calculating the SEM and SDC were to have been used.

There are, however, also limitations to this study. Although the researchers who carried out the individual translation and validation studies received the same guidance on the preferred design and conduct of these studies, local circumstances sometimes led them to deviate with regards to measurement of sarcopenia components (muscle mass, muscle strength and physical performance). Therefore, the methods for establishing the presence of sarcopenia are not standardized. This could, however, also be regarded as an opportunity in that we have a mix of subjects in the combined sample that represent a spectrum of methods and instruments. Secondly, because of the original purpose of the included studies, only the SarQoL questionnaire was administered twice, to calculate the test-retest reliability. It would have been preferable to compare the SEM and SDC of the SarQoL questionnaire to values for the SF-36 and the EQ-5D measured in the same populations. But, since this data does not exist, we feel that a comparison to data from the literature was the second-best option and does provide a valid frame of reference.

## Conclusion

The current study, which analyzed a sample of 278 subjects from 9 validation studies, obtained a standard error of measurement of 2.65 points and a smallest detectable change of 7.35 points for the Overall score of the SarQoL questionnaire. These values can be applied in future longitudinal research to evaluate the veracity of measured changes.

## Supporting information

S1 Table One-way Anova (Tukey) for age. (PDF)

S2 Table One-way Anova (Tukey) for BMI. (PDF)

S3 Table One-way Anova (Tukey) for number of drugs. (PDF)

S4 Table One-way Anova (Tukey) for number of concomitant illnesses. (PDF)

S5 Table Chi-squared test for gender [n(%)]. (PDF)
