# Supplementary material for: Standard error of measurement and smallest detectable change of the Sarcopenia Quality of Life (SarQoL) questionnaire: An analysis of subjects from 9 validation studies
Source: PLoS One. 2019 Apr 29;14(4):e0216065. doi: 10.1371/journal.pone.0216065 (PMC6488089; doi:10.1371/journal.pone.0216065)
Supplement: S1 Table — (PDF) [file pone.0216065.s001.pdf]

Anton Geerinck
Anton.geerinck@uliege.be

Table S1: One-way Anova (Tukey) for Age									
	Belgium (Dutch)	Belgium (French)	Brazil	Czech Republic	England	Lithuania	Greece	Poland	Spain
Belgium (Dutch)	1								
Belgium (French)	0.382	1							
Brazil	<0.001	0.124	1						
Czech Republic	0.950	0.005	<0.001	1					
England	0.995	0.997	0.093	0.696	1				
Lithuania	1.000	0.469	<0.001	0.426	1.000	1			
Greece	<0.001	0.038	0.999	<0.001	0.073	<0.001	1		
Poland	0.002	0.629	0.909	<0.001	0.461	0.001	0.969	1	
Spain	0.805	1.000	0.154	0.132	1.000	0.913	0.107	0.667	1
