# Supplementary material for: Standard error of measurement and smallest detectable change of the Sarcopenia Quality of Life (SarQoL) questionnaire: An analysis of subjects from 9 validation studies
Source: PLoS One. 2019 Apr 29;14(4):e0216065. doi: 10.1371/journal.pone.0216065 (PMC6488089; doi:10.1371/journal.pone.0216065)
Supplement: S2 Table — (PDF) [file pone.0216065.s002.pdf]

Anton Geerinck
Anton.geerinck@uliege.be

Table S2: One-way Anova (Tukey) for BMI									
	Belgium (Dutch)	Belgium (French)	Brazil	Czech Republic	England	Lithuania	Greece	Poland	Spain
Belgium (Dutch)	1								
Belgium (French)	0.030	1							
Brazil	0.917	0.949	1						
Czech Republic	0.223	<0.001	0.025	1					
England	0.663	1.000	1.000	0.007	1				
Lithuania	0.395	0.796	1.000	<0.001	1.000	1			
Greece	0.132	0.989	1.000	<0.001	1.000	0.998	1		
Poland	1.000	0.008	0.806	0.334	0.496	0.165	0.038	1	
Spain	0.565	0.997	1.000	0.001	1.000	1.000	1.000	0.370	1
