# Supplementary material for: Standard error of measurement and smallest detectable change of the Sarcopenia Quality of Life (SarQoL) questionnaire: An analysis of subjects from 9 validation studies
Source: PLoS One. 2019 Apr 29;14(4):e0216065. doi: 10.1371/journal.pone.0216065 (PMC6488089; doi:10.1371/journal.pone.0216065)
Supplement: S3 Table — (PDF) [file pone.0216065.s003.pdf]

Anton Geerinck
Anton.geerinck@uliege.be

Table S3: One-way Anova (Tukey) for number of drugs									
	Belgium (Dutch)	Belgium (French)	Brazil	Czech Republic	England	Lithuania	Greece	Poland	Spain
Belgium (Dutch)	1								
Belgium (French)	0.000	1							
Brazil	0.001	0.999	1						
Czech Republic	0.001	0.996	0.934	1					
England	0.234	0.996	0.946	1.000	1				
Lithuania	0.986	0.000	0.004	0.001	0.521	1			
Greece	1.000	0.000	0.000	0.000	0.059	0.615	1		
Poland	0.710	0.000	0.000	0.000	0.005	0.048	0.867	1	
Spain	0.722	0.457	0.331	0.785	0.993	0.969	0.311	0.032	1
