# Supplementary material for: Standard error of measurement and smallest detectable change of the Sarcopenia Quality of Life (SarQoL) questionnaire: An analysis of subjects from 9 validation studies
Source: PLoS One. 2019 Apr 29;14(4):e0216065. doi: 10.1371/journal.pone.0216065 (PMC6488089; doi:10.1371/journal.pone.0216065)
Supplement: S4 Table — (PDF) [file pone.0216065.s004.pdf]

Anton Geerinck
Anton.geerinck@uliege.be

Table S4: One-way Anova (Tukey) for number of concomitant illnesses									
	Belgium (Dutch)	Belgium (French)	Brazil	Czech Republic	England	Lithuania	Greece	Poland	Spain
Belgium (Dutch)	1								
Belgium (French)	<0.001	1							
Brazil	0.035	0.821	1						
Czech Republic	<0.001	0.236	0.022	1					
England	NA	NA	NA	NA	1				
Lithuania	0.861	<0.001	0.214	<0.001	NA	1			
Greece	0.900	<0.001	0.208	<0.001	NA	1.000	1		
Poland	0.386	<0.001	<0.001	<0.001	NA	0.002	0.003	1	
Spain	0.136	0.270	0.998	<0.001	NA	0.576	0.561	<0.001	1
