# Supplementary material for: Standard error of measurement and smallest detectable change of the Sarcopenia Quality of Life (SarQoL) questionnaire: An analysis of subjects from 9 validation studies
Source: PLoS One. 2019 Apr 29;14(4):e0216065. doi: 10.1371/journal.pone.0216065 (PMC6488089; doi:10.1371/journal.pone.0216065)
Supplement: S5 Table — (PDF) [file pone.0216065.s005.pdf]

Anton Geerinck
Anton.geerinck@uliege.be

Table S5: Chi-squared test for gender [n(%)]										
	Belgium (Dutch)	Belgium (French)	Brazil	Czech Republic	England	Lithuania	Greece	Poland	Spain	p-value Chi²
n	26	29	12	48	10	58	50	30	15	
Gender										
Male	14 (53.8)	10 (34.5)	6 (50.0)	11 (22.9)	7 (70.0)	30 (51.7)	13 (26.0)	5 (33.3)	5 (33.3)	0.009
Female	12 (46.2)	19 (65.5)	6 (50.0)	37 (77.1)	3 (30.0)	28 (48.3)	37 (74.0)	19 (63.3)	10 (66.7)	
